# Familial Association of Granulomatosis With Polyangiitis: A Case-Based Review of Literature

**DOI:** 10.7759/cureus.40786

**Published:** 2023-06-22

**Authors:** Larabe Farrukh, Aqsa Mumtaz, Faria Sami, Maria Faraz, Khoa Richard Ngo

**Affiliations:** 1 Internal Medicine, Albany Medical Center, Albany, USA; 2 Internal Medicine, King Edward Medical University, Lahore, PAK; 3 Internal Medicine, Allama Iqbal Medical College, Lahore, PAK; 4 Pathology, Albany Medical Center, Albany, USA; 5 Rheumatology, Albany Medical Center, Albany, USA

**Keywords:** granulomatosis with polyangiitis (gpa), familial disease, genetic syndromes, anca associated vasculitis, auto immune

## Abstract

Anti-neutrophil cytoplasm antibody (ANCA)-associated vasculitis (AAV) is a class of small vessel vasculitis that includes granulomatosis with polyangiitis (GPA), eosinophilic GPA (EGPA), and microscopic polyangiitis (MPA). Despite extensive research, the mechanisms behind AAV etiology remain obscure. The genetics of AAV is a complex area of investigation because of the rarity of familial cases. However, recent multi-center genome-wide association studies (GWAS) have greatly contributed to our understanding of the genetic basis of AAV. In this study, we report a rare occurrence of GPA in two Caucasian family members who presented with similar clinical symptoms and performed a comprehensive review to study the present literature available regarding the heritability of this disease.

## Introduction

Anti-neutrophil cytoplasm antibody (ANCA)-associated vasculitis (AAV) is a type of small vessel vasculitis characterized by inflammation of both arteries and veins. AAV includes granulomatosis with polyangiitis (GPA), eosinophilic GPA (EGPA), and microscopic polyangiitis (MPA). GPA is a rare necrotizing vasculitis often affecting the upper and lower respiratory tract and the kidneys. Diagnosis is based on characteristic clinical and pathological findings, usually in the presence of ANCA directed against neutrophil and monocyte proteinase 3 (PR3) or myeloperoxidase (MPO) [1,2].

Despite extensive research, the mechanism behind AAV etiology remains obscure [3]. There seems to be a complex interplay between genetic and environmental factors that are not fully understood yet. The genetics of AAV is a complex area of research because of the rarity of familial cases. However, recent multi-center genome-wide association studies (GWAS) have greatly contributed to our understanding of the genetic basis of AAV. It has been observed that the AAV subtypes are associated with distinct human leukocyte antigen (HLA) variants, i.e., GPA with HLA-DP1, MPA with HLA-DQ, and eosinophilic GPA with HLA-DRB4 [3,4].

While familial aggregation has been reported in other autoimmune diseases, including rheumatoid arthritis and systemic lupus erythematosus, there have been very few cases of GPA clustering in families. It has been shown that these family members shared an identical HLA haplotype, i.e., HLADPB1 allele, which has been linked to GPA in previous studies as well [5,6].

## Case presentation

Case 1

The patient was a 62-year-old female who presented initially in 2018 with the complaint of bilateral lower extremity pain, ulcerations, erythema, and swelling. She had been experiencing polyarthralgia mainly involving small joints of the fingers, scattered petechial/purpuric rash on the upper and lower extremities, and black discoloration of fingertips. She was started on methotrexate by an outpatient rheumatologist for suspected inflammatory arthritis and had a pending autoimmune workup.

**Figure 1:**
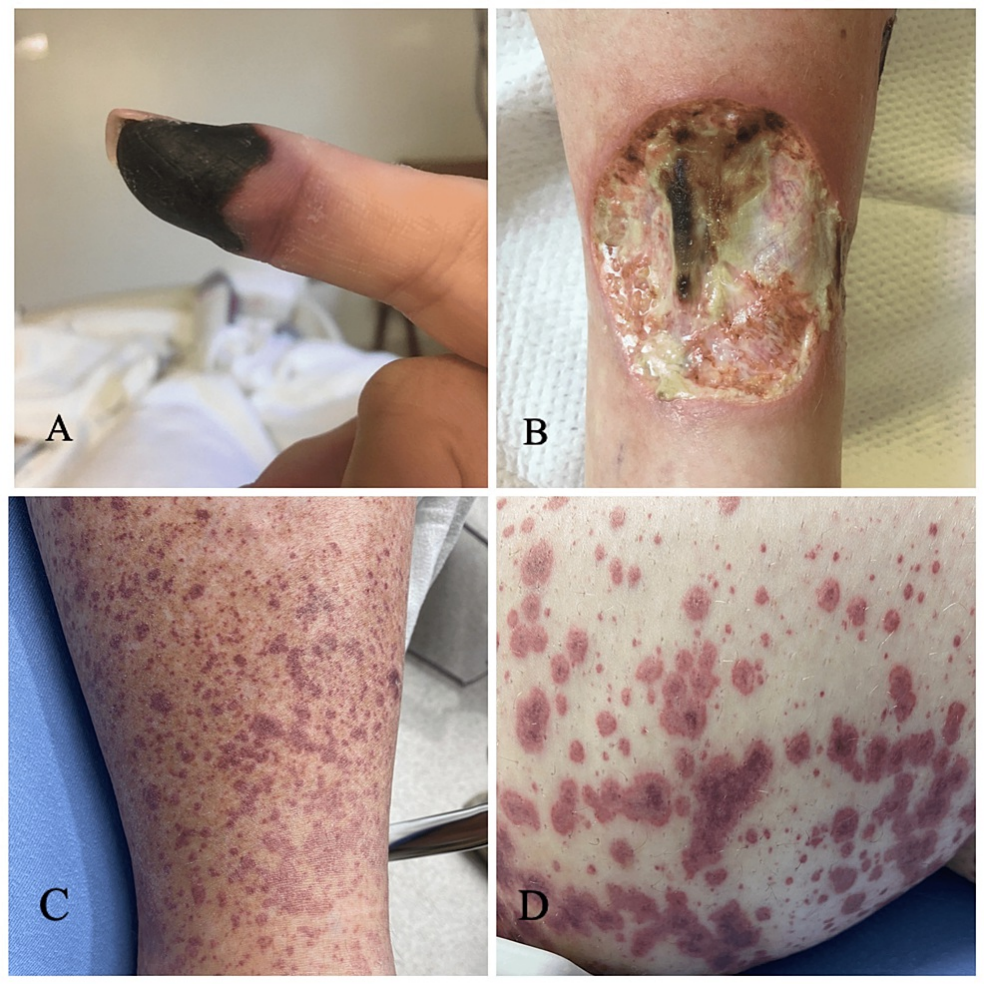
A and B showing fingertip necrosis and pyoderma gangrenosum in patient 1. C and D show palpable purpura in patient 2.

On presentation, she was vitally stable. A physical exam revealed black discoloration with areas of necrosis on the fingertips. She had blanchable erythema on the hands and feet, with areas of necrosis on the tips of her toes (Figure 1). The patient had extensive ulceration (pyoderma gangrenosum) on the right shin measuring 3 x 3 cm and the left shin measuring up to 3 x 2 cm with surrounding erythema. She was admitted for possible autoimmune vasculitis and rheumatology was consulted.

Investigations

Laboratory investigation showed an elevated WBC count of 12.6 x 10*3/uL (normal 4.1-9.3 x 10*3/uL) and low hemoglobin of 10.8 g/dL (normal 11-14.7 g/dL). Renal function and urinalysis were normal. Erythrocyte sedimentation rate (ESR) was elevated at 60 mm/hr (normal 0-25 mm/hr). Serology showed elevated titers of antinuclear antibody (ANA) 160 with indirect immunofluorescence showing a speckled pattern (normal < 160), cytoplasmic ANCA of 320 (normal <20), and anti-proteinase-3 of 51.3 (normal <20). Rheumatoid factor was elevated to 62 IU/ml (normal <14 IU/ml). Perinuclear ANCA, atypical ANCA, and anti-MPO were normal. An autoimmune panel including anti-centromere Ab, anti-RNA polymerase III Ab, anti-Scl-70 Ab, Sjogren's panel including anti-SSA/SSB, anti-phospholipid Ab, anti-cardiolipin Ab, anti-Beta-2 glycoprotein Ab, anti-CCP Ab, anti-glomerular Ab were negative. The patient had normal complement levels and negative cryoglobulins. HIV and hepatitis B/C screens were negative (Table 1).

**Table 1:** Laboratory workup WBC: white blood cell, ESR: erythrocyte sedimentation rate, ANA: antinuclear antibody

LABS	NORMAL VALUE	PATIENT 1	PATIENT 2
WBC count	4.1-9.3 x 10*3/uL	12.6	13.3
Hemoglobin	11-14.7 g/dL	10.8	11.9
ESR	0-25 mm/hr	60	64
ANA	Titer < 160	160 (speckled pattern)	Negative
Cytoplasmic ANCA	Titer <20	320	160
Anti-proteinase-3 Ab	Titer <20	51.3	375
Rheumatoid factor	Titer <14 IU/ml	62	108

**Figure 2:**
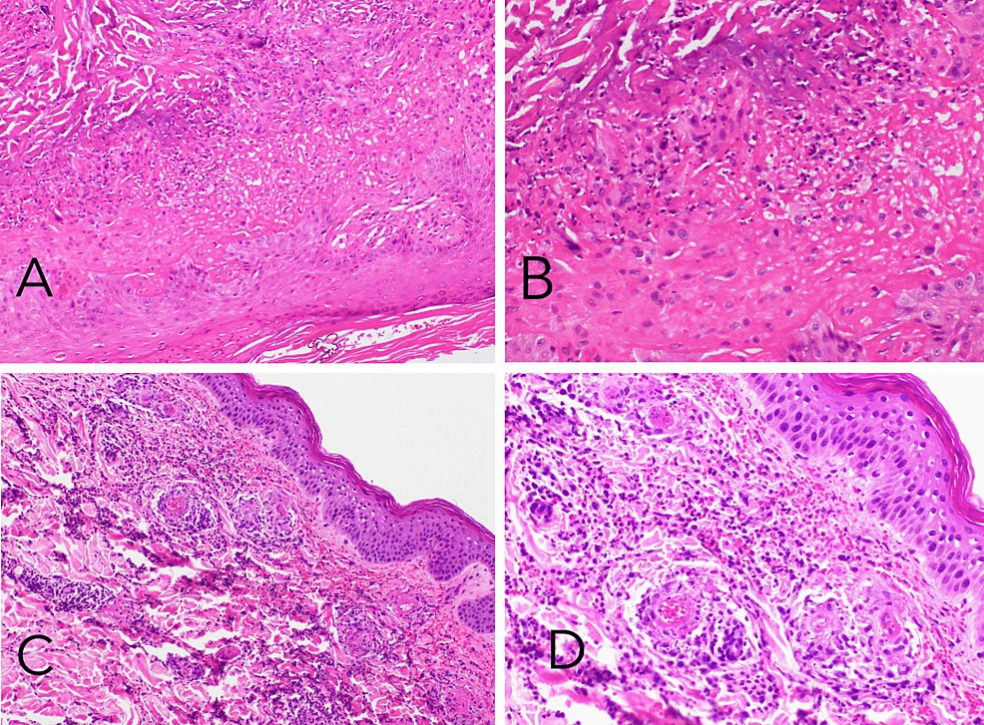
Slides A and B - Hematoxylin and Eosin-stained sections show palisaded neutrophilic granulomatous dermatosis in Patient 1 (10X and 20X). Slides C and D - Hematoxylin and Eosin stained sections show a superficial to deep dermal perivascular infiltrate of conspicuous neutrophils, occasional surface, karyorrhexis, and red blood cell extravasation with a prominent fibrinoid change of vessel walls in Patient 2 (10X and 20X).

Skin punch biopsy showed palisaded neutrophilic granulomatous dermatosis, which can be identified in primary systemic vasculitis (Figure 2). She also had a right lower lobe bronchoalveolar lavage showing hemosiderin-laden macrophages consistent with diffuse alveolar hemorrhage. In light of these findings, the patient was diagnosed with GPA.

Treatment

The patient underwent wound debridement multiple times for extensive lower extremity ulcers. She was started on oral prednisone and weekly rituximab infusion, with follow-up at the rheumatology clinic. She had multiple hospital visits for uncontrolled pain in her lower extremities. She continues to receive regular rituximab infusions and is currently in remission.

Case 2

The patient was a 32-year-old male with a past medical history of chronic sinusitis, inflammatory arthritis, and major depressive disorder who presented to the hospital with the complaint of cough with worsening hemoptysis, dyspnea, bilateral lower extremity purpuric rash, and polyarthralgia.

According to the patient, his symptoms started five months ago. Initially, he developed intermittent pain and swelling in the small joints of the hand and bilateral wrists. He was found to have an elevated rheumatoid factor and was started on a tapering course of steroids, which did not improve his symptoms. This was followed by the appearance of a purpuric rash and peripheral edema in his bilateral lower extremities. Before the presentation, he also developed a productive cough with hemoptysis.

On presentation, the patient was found to be tachycardic and hypoxic with an oxygen saturation of 89%. Physical exam showed crepitations in bilateral lung fields, 1+ pitting edema in bilateral lower extremities, tenderness in L shoulder and wrists, and swelling of the metacarpophalangeal and proximal interphalangeal joints. He had a diffuse palpable purpuric rash extending from his feet up to the level of the umbilicus, with areas of confluence and ecchymosis (Figure 1). A review of systems was positive for fatigue, night sweats, bilateral nasal ulcers, congestion, dyspnea, and polyarthralgia.

Investigations

Initial laboratory investigation showed an elevated WBC count of 13.3 (normal 4.1-9.3 x 10*3/uL), elevated ESR 64 (normal 0-14 mm/hr), and CRP 156 (normal <8 mg/L). Renal function was normal. Urinalysis showed 1+ protein, 1+ leukocyte esterase, 3+ hemoglobin, and 10-20 RBCs per hpf. Chest x-ray showed bilateral nodular opacities in mid and lower lung fields. This was followed by a CTA chest which showed centrilobular nodular opacities in bilateral lung fields concerning intra-alveolar bleeding. Rheumatology was consulted due to concerns about an autoimmune/vasculitic process. The patient was started on oral prednisone, pending further workup (Table 1).

Further labs showed normal complement levels, IgA levels, and negative cryoglobulins. Serology showed negative ANA, anti-double-stranded DNA, anti-Smith antibodies, anti-SSA/SSB, early Sjogren's panel, anti-RNP, anti-cardiolipin, beta-2 glycoprotein, and anti-CCP. Perinuclear ANCA, atypical ANCA, and anti-MPO were normal. The titer of cytoplasmic ANCA was 160 (normal <20) and anti-proteinase-3 was 375.1 (normal <20). Rheumatoid factor was elevated to 108 (normal <14 IU/ml). Hepatitis B and C panels were negative.

Bronchoalveolar lavage was done in the setting of worsening hemoptysis which retrieved bloody fluid; however, cultures were negative. Skin punch biopsy hematoxylin and eosin stained sections showed superficial to deep dermal perivascular infiltrate of neutrophils, occasional eosinophils, karyorrhexis, and red blood cell extravasation with a prominent fibrinoid change of vessel wall. Direct immunofluorescence showed deposition of IgM and C3, C5b, and C4d with some fibrinogen in superficial dermal vessels consistent with leukocytoclastic vasculitis (Figure 2).

In light of these findings (history of sinusitis, nasal ulcers, palpable purpura, hemoptysis, and diffuse pulmonary nodules suggestive of diffuse alveolar hemorrhage) and family history of GPA, the patient was diagnosed with an autoimmune-mediated small vessel vasculitis, with the leading differential being GPA. HUV was ruled out in the setting of normal complement levels (C1q, C3/C4), negative ANA, and Sjogren's panel. Others on the differential, but less likely include EGPA which was ruled out in the absence of eosinophilia, normal IgE levels, negative p-ANCA/anti-MPO Ab, or clinical features like asthma and neuropathy. Cryoglobulinemic vasculitis was ruled out in the absence of cryoglobulins.

Treatment

The patient was started on a pulse dose of steroids. He had worsening hypoxic respiratory failure, requiring intubation. The plan was to initiate rituximab or cyclophosphamide along with plasmapheresis. However, the patient quickly deteriorated in the ICU. He went into sustained ventricular tachycardia followed by pulseless electrical activity arrest and died on the sixth day of hospitalization despite aggressive resuscitation efforts.

## Discussion

Literature review

Methods

In this study, we report a rare occurrence of GPA in two Caucasian family members, a mother and her son who presented with similar clinical symptoms and performed a comprehensive literature review.

A literature search was conducted on PubMed, Cochrane, and Google Scholar from inception to December 2022 using Medical Subject Headings (MeSH) terms for "granulomatosis with polyangiitis," "ANCA vasculitis," and "familial." The search strategy combined free text search, MeSH terms, and synonyms for identifying observational studies, case series, and case reports. We also searched for additional articles from the reference list of the original articles.

Eligibility Criteria

We included case reports, case series, and observational studies which reported the familial cases of GPA.

Study Selection

The authors screened the titles and abstracts of all references which were identified by the literature search and excluded irrelevant studies. We read the full texts of the remaining articles and included the studies which met the inclusion criteria.

Data Extraction

The authors extracted data from the included studies using a pre-specified data collection form in Microsoft Excel. Data about the study year, patient characteristics, serological profile, diagnosis, treatment, and outcome were extracted.

Results

A total of 8 studies and 17 patients were included. The mean age was 39.2 years, sex distribution was 58.8% (n=10) males and 41.1% (n=7) females. About 88.2% of the cases were diagnosed with GPA (n=15); however, EGPA was found in 5.8% (n=1) and MPA in 5.8% (n=1) of the familial cases. Serological results were diverse, and out of 13 patients, 38.4% (n=5) were c-ANCA positive, 23% (n=3) had p-ANCA, 23% (n=3) had anti-MPO Ab, and 30 % had anti-PR3 Ab(n=4). Twenty-three percent of the patients were positive for ANCA (n=3) without specifying whether c-ANCA or p-ANCA. Serological results were not reported for 4/17 patients. Out of the total 17 patients, 15 received treatment. About 93.3% (n=14) were treated with steroids, 86.6% (n=13) received cyclophosphamide, 53.3% (n=8) received azathioprine, 13.3% (n=2) received rituximab, 6.6% (n=1) received methotrexate, and 6.6% (n=1) received plasmapheresis. After the treatment phase, all patients survived, 35.3% (n=6) went into remission, while 17.64 % relapsed (n=3) (Table 2).

**Table 2:** Characteristics of patients included in the study

Study	Year	No. of patients	Relation	Age (Y)	Sex	Serology	Diagnosis	Treatment	Outcome	HLA type
Muniain et al. [7]	1986	2	Siblings	20	F	NA	GPA	Steroids, CYC	Alive	N/A
				24	F	NA	GPA	Refused	Alive	N/A
Tanna et al. [10]	2012	3	Brother	33	M	c-ANCA, PR-3	GPA	Steroids, CYC, AZA	Alive	HLA-A 68, B44, Cw7, DR 17, 52, DQ2. HLA-DPB1*0401
			Sister	33	F	PR-3	GPA	Steroids, CYC, AZA	Alive, remission	HLA-A 68, B44, Cw7, DR 17, 52, DQ2. HLA-DPB1*0401
			Father	NA	M	NA	GPA	Steroids, AZA	Alive	HLA-A 68, B44, Cw7, DR 17, 52, DQ2. HLA-DPB1*0401
Prendecki et al. [11]	2016	2	Siblings	49	M	c-ANCA, PR-3	GPA	Steroids, CYC, PLEX, RTX, AZA	Alive, remission	HLA A*3, B*44, Cw7,DR*11, DQ*7, DQB1*03/05,
				56	M	p-ANCA, MPO	MPA	Steroids, CYC, RTX, AZA	Alive, remission	HLA A*3, B*44, Cw7, DR*11, DQ*7, DQB1*03/05,
Hay et al. [12]	1991	2	Siblings	57	M	ANCA	GPA	Steroids, AZA	Alive, remission	HLA A3,10, B8,17, DR 3, 7
				49	F	ANCA	GPA	Steroids, CYC, HD	Alive, relapsed	HLA A3, 10, B8, DR3, 4
Sewell et al. [13]	1990	2	Mother	63	F	c-ANCA	GPA	Steroids, CYC, PLEX	Alive	HLA A9, Al 1, B14, B18, DR1, DR2
			Daughter	38	F	c-ANCA	GPA	CYC	Alive	HLA A2, A9, B12, B14, Bw4, Bw6, DR1
Hull et al. [15]	2000	2	Daughter	8	F	p-ANCA, MPO	GPA	Steroids, CYC, AZA,	Alive, ESRD	HLA A24, B35 DR B4, DQB3
			Father	NA	M	p-ANCA, MPO	GPA	NA	Alive ESRD	HLA A24, B35 DR B4, DQB3
Manganelli et al. [16]	2003	2	Father	54	M	ANCA Negative	EGPA	Steroids, CYC	Alive, remission	HLA A*03; B*07; C*w07; DRB1*0404, DQB1*0302.
			Son	33	M	c-ANCA, PR-3	GPA	Steroids, CYC. MTX	Alive, remission	HLA A*03; B*07; C*w07; DRB1*0404, DQB1*0302.
Stoney et al. [17]	1991	2	Siblings	42	M	ANCA	GPA	Steroids, CYC	Alive, relapsed	HLA-A3, A31; Bw60, B14; DR4, DRw6
				30	M	NA	GPA	Steroids, CYC, AZA	Alive, relapsed	HLA-A11, A31; Bw60, B44; DR4, DRx

Discussion

Family recurrence is a strong indicator of a possible heritability in autoimmune disorders. Familial clusters can be caused by chance, genetic susceptibility, environmental triggers, or a combination of all combined. Muniain et al. with their report of limited GPA occurring in two sisters spiked a debate on the familial occurrence of the disease [7]. Nagibov et al. on the other hand described an unusual case of GPA in a couple: a husband who succumbed to the disease and a wife who developed GPA later on. This led to an important discussion about whether environmental triggers also play an important role in the manifestation of ANCA-associated vasculitis [8]. This was further supported by Weiner et al. who reported about identical twins who were discordant for GPA [9].

Recently, there have been a few more reports of familial AAV favoring a genetic pattern. These studies describe familial clusters of patients presenting at different times, both with similar and different phenotypes and ANCA sub-type. Tanna et al. presented three cases in an Indo-Asian family. The first case was a 33-year-old male who presented with nasal congestion and deafness. Serology was positive for c-ANCA and anti-PR3. The patient was diagnosed with GPA. Later, the patient's sister presented with supraorbital pain, sinusitis, hearing loss, and nasal congestion. She underwent a nasal biopsy which confirmed GPA and was found to be PR3-ANCA positive. Retrospectively, it was discovered that their father had also developed rapidly progressive glomerulonephritis associated with GPA in the past, requiring a renal transplant. The three family members shared a remarkably similar disease phenotype. HLA typing demonstrated a common haplotype: HLA-A 68, B44, Cw7, DR 17, 52, and DQ2 [10].

Similarly, Prendecki et al. presented the case of two brothers presenting with AAV with varying clinical syndromes and different ANCA specificities. The first patient presented with uveitis, episcleritis, and skin rash. He was found to have renal and pulmonary involvement. Serology showed c-ANCA and anti-PR3 positivity, making a diagnosis of GPA. Treatment was initiated and the patient went into remission. Six months later, the patient's brother presented with acute kidney injury. He was found to be p-ANCA and anti-MPO positive. Renal biopsy showed pauci-immune crescentic glomerulonephritis. Surprisingly, he was diagnosed with MPA and treated with steroids, rituximab, and cyclophosphamide, which resulted in remission [11]. Similarly, Hay et al. reported on two siblings with GPA, both with renal and pulmonary involvement [12]. Sewell and Hamilton reported a mother and daughter with GPA, both with renal and ENT involvement. Knudsen et al. reported a family of eight siblings, two of whom got independently diagnosed with GPA and one healthy sibling positive for ANCA without any disease manifestation [13-17].

Two genome-wide association studies (GWAS) have been performed to identify the genetic factors associated with AAV. The first GWAS in a European population in 2012 demonstrated that HLA-DP rs3117242 (G) was the strongest signal in the HLA region [3]. Another GWAS in Canadian and American populations conducted in 2017 showed that SNPs rs141530233 and rs1042169 at HLA-DPB1 had the largest effect on the risk of developing AAV. These studies not only indicated a significant association between AAV and HLA regions but also showed genetic distinctions between different clinical phenotypes and ANCA specificity. GPA and PR3-ANCA AAV are associated with HLA-DPB1 and HLA-DPA1, while MPA and MPO-ANCA AAV are associated with HLA-DQB1 and HLA-DQA2 [18].

Another GWAS for GPA with 492 patients and 1,506 healthy individuals of European descent identified 32 SNPs across the HLA region; among them, HLA-DPB1 rs9277554 and HLA-DPA1 rs9277341 were significantly associated with GPA. In another study with 150 GPA patients and 100 healthy controls conducted in northern Germany, HLA-DPB1*0401 was identified to be associated with GPA, and DPB1*0401/RXRB03 haplotype frequency was significantly increased in patients with GPA [19,20]. GWAS also revealed that polymorphic variants of certain genes encoding proteinase 3-PR3 (the antigenic target of ANCA in GPA) and its main inhibitor, alpha-1 antitrypsin, are highly associated with GPA and more significantly with PR3-ANCA positivity [21,22].

It is crucial to acknowledge that a subset of patients with GPA may test negative for ANCA. These ANCA-negative cases are more commonly observed among individuals with limited disease. Therefore, it is imperative to thoroughly review the complete classification criteria before reaching a final diagnosis [23].

## Conclusions

The etiology behind the mechanics of AAV, specifically GPA, remains obscure; however, recent studies and case reports have predicted a genetic component to play a pivotal role. Genetic testing, HLA subtyping, and further studies are warranted to determine the possible hereditary transmission of the disease.

In summary, these findings could help elucidate the etiology of AAV and develop new biomarkers for early diagnosis and targeted therapy. Although very informative, all these findings represent only the beginning of a new exciting, and dynamic phase in this field. Furthermore, information on the possible heritability of AAV is of clinical importance because family members would often want to know whether having AAV puts their closest relatives at increased risk of developing the disease.
